# A social media competitive intelligence framework for brand topic identification and customer engagement prediction

**DOI:** 10.1371/journal.pone.0313191

**Published:** 2024-11-25

**Authors:** Xingting Ju

**Affiliations:** Department of Marketing & Strategy, Rabat Business School, International University of Rabat, Rabat-Salé, Morocco; Oakland University, UNITED STATES OF AMERICA

## Abstract

**Purpose:**

The COVID-19 pandemic has changed customer social media engagement behavior, which challenges the establishment of effective marketing strategies to strengthen digital communication with customers and leads to new opportunities for social media competitive intelligence analytics. This study presents a new social media competitive intelligence framework that incorporates not only the detection of brand topics before and during the COVID-19 pandemic but also the prediction of customer engagement.

**Design/Methodology/Approach:**

A sector-based empirical study is conducted to illustrate the implementation of the proposed framework. We collected tweets generated by 23 leading American catering brands before and during the pandemic. First, we used Amazon Comprehend and Latent Dirichlet allocation (LDA) to extract sentiments and topics behind unstructured text data. Second, we trained and compared the performance of six machine learning algorithms to find the optimal classifiers.

**Findings:**

The study reveals significant shifts in social media engagement topics following the COVID-19 pandemic. Pre-pandemic topics primarily included “Food and lifestyle”, “Promotion”, “Food ordering”, “Food time”, and “Food delivery”. During the pandemic, the topics expanded to include “Social responsibility” and “Contactless ordering”. For predicting customer engagement, the performance metrics show that Random Forest and C5.0 (C50) are generally the best-performing models, with Random Forest being particularly strong for "Likes" and “Retweets”, while C50 performs best for “Replies”.

**Originality:**

This framework differentiates itself from existing competitive intelligence frameworks by integrating the influence of external factors, such as the COVID-19 pandemic, and expanding the analysis from topic detection to customer engagement prediction. This dual focus provides a more comprehensive approach to social media competitive intelligence.

## 1. Introduction

Competitive intelligence is a process that includes planning, collection, analysis, and dissemination of information [1]. The wide adoption of social media is an essential source of competitive intelligence [1, 2]. By analyzing data generated on social media, firms can track competitors’ activities [3] and monitor their communication mode with customers [4]. This information is then transformed into actionable insights, facilitating the prediction of consumer behavior, strategy development, and decision-making processes. These processes are particularly critical during extreme events or crises [1].

The outbreak of COVID-19 pandemic represents one of the most significant environmental changes in the modern marketing history [5], which affects customer social media engagement behaviors [6, 7]. Customer engagement in social media is defined as customers’ behavioral manifestations beyond purchasing in response to brand-related content in social media [8, 9]. Firms face the challenge of establishing effective marketing strategies to strengthen digital communication with customers in uncertain environments and when customers’ perceptions and attitude towards the brand have changed [5, 10, 11]. Customers’ perception and attitude may also change due to the actions that firms take during extremely turbulent periods [5, 10]. Unique situations provide opportunities for social media competitive intelligence analytics, which allow firms to identify competitors’ social media activities and predict customer engagement [10]. Such forecasts enable firms to gauge the effectiveness of their social media marketing efforts, adapt strategy to the changes, and be prepared for possible future events [12, 13].

The social media competitive intelligence literature provides frameworks that integrate several data analysis methods to guide marketers for gaining actionable knowledge from social media data, including sentiments [2, 13–19] and topics [2, 20]. Such knowledge is further applied for supporting decision-making [14], social media promoted post detection [21], firms’ social media marketing level prediction [13], firm performance [19], and operations and service management [2]. However, important research gaps remain.

First, although changes in customer engagement behavior require an adaptation of marketing practices [10], investigations on how firms respond to this type of changes are still scarce. While researchers have revealed the topic evolution of the tweets published by the fortune CEOs before and during the COVID-19 pandemic [22], there are no studies comparing the impact of the pandemic at the brand level. Unlike posts from CEOs, which focus on firms’ strategic level [22], posts from brand-hosted social media accounts focus more on firms’ operational level that strengthens the relationship with customers [23], providing customer service, product information, special offers, and various types of entertainment [24].

Second, competitive intelligence frameworks suggest firms the way to incorporate unstructured data into the data analysis, which allows them to predict what will happen in their competitive environment [25]. Researchers have conducted studies using firm-generated content (FGC) as input features and have sorted the optimal machine learning algorithms to predict types of brand personality [26], brand promoted posts [21], and firms’ social media engagement level [13]. Despite customer engagement prediction enables firms to clearly set their marketing goals for their posts and choose appropriate marketing strategies to engage with customers [27], efforts to incorporate the prediction of customer engagement in social media competitive intelligence frameworks are scarce. While existing research focuses on the prediction of customer engagement as a whole concept [13], the prediction of different types of engagement on social media platforms, including likes, retweets, and replies [28] has not been discussed in the literature. The prediction of different types of customer engagement is important because it allows brands to tailor their strategies more effectively. Understanding the nuances of how customers engage—whether they are more likely to like, retweet, or reply—can help companies optimize their content for higher interaction rates, improve customer satisfaction, and ultimately drive better business outcomes [28].

To address the gaps mentioned above, this conceptual and empirical research has three objectives: (i) to propose a new social media competitive intelligence framework with the phases of brand topic detection and customer engagement prediction, (ii) to explore firms’ topic patterns at the brand level before and during the COVID-19 pandemic and (iii) to train classifiers that most accurately predict the three types of customer engagement—like, retweet, and reply—in response to FGC on social media. Unlike existing frameworks, which typically focus on extracting knowledge such as sentiment or topics from social media data, our framework integrates the prediction of customer engagement behaviors. This is crucial because the ability to predict distinct engagement actions (likes, retweets, replies) allows firms to tailor their content and strategies more effectively, a feature that is often missing in current competitive intelligence models. To validate and illustrate the implementation of the proposed framework, we apply a sector-based analysis to 23 leading catering brands in the United States, a sector significantly impacted by the COVID-19 pandemic.

This study makes both theoretical and practical contributions. From a theoretical perspective, the research fills the void left by existing frameworks by incorporating (i) the influence of external environmental changes, such as the pandemic, on brand topics, and (ii) the ability to predict customer engagement at a granular level. These features address gaps in the literature where most frameworks fail to consider how sudden environmental disruptions affect brand communication and customer responses. From a practical perspective, our framework provides marketing practitioners with actionable insights into how customer engagement can be predicted and adjusted in real-time. This allows firms to more effectively adapt their social media strategies and marketing goals in response to changing environments, ultimately driving better outcomes in uncertain market conditions.

## 2. Literature review on social media competitive intelligence frameworks

Competitive intelligence is defined as a process that helps organizations to collect information from customers, competitors, and the business environment before transforming it into knowledge which serves to support decision-making [1]. The rise of social media provides opportunities for information collection and dissemination, content generation, and interactive communication [29]. In the social media context, competitive intelligence is termed social media intelligence [30] or social media competitive intelligence [15, 31] as we refer in this paper. Researchers have proposed several frameworks to describe how to gain knowledge by mining social media data. To systematically review the social media intelligence literature, we searched for journal articles in ScienceDirect, Scopus, and Web of Science using the following keywords in the title, abstract, keywords, and full text of the articles: “social media analytics”, “social media competitive intelligence”, “social media business intelligence”, and “social media big data analysis.” We identified 11 relevant studies. Table 1 in the online material summarizes the objectives, industries, methods, and contributions of the studies, and we briefly present them in the next paragraph.

**Table 1:** Social media competitive intelligence frameworks.

Authors	Objective	Industry	Method		Contribution
			Data collection	Data analysis	
[31]	To explore how to perform a social media competitive intelligence analysis and transform data into knowledge for decision makers.	Pizza	User-generated content on Facebook and Twitter from the three largest pizza chains brands was collected.	Case study. Themes analysis.	By comparing customer engagement patterns of different brands in Facebook and Twitter, the study highlights the value of social media data for decision making at the industry level.
[15]	To propose a framework for social media competitive intelligence to enhance business value.	Retailing	User-generated content on Twitter from two largest retail chains brands was collected using Twitter API.	Case study. Sentiment analysis.	The study proposes a framework of social media competitive intelligence to illustrate how firms can generate tangible and intangible business value by leveraging social media analytics.
[16]	To propose a social media competitive analytics framework with sentiment benchmarks for gaining industry-specific marketing intelligence.	Retailing	User-generated content on Facebook, Twitter, forums, blogs, and other platforms from five retail brands was collected using APIs, RSS, HTML parsing, and manual copying.	Sentiment analysis and themes analysis.	The study develops a novel social media competitive analytics framework with sentiment benchmarks to shed light on how to enhance marketing intelligence and generate business insight reports through various social media competitive analysis.
[17]	To develop a framework to explore how social media competitive analytics is applicable for understanding customer experiences.	Retailing	User-generated content on Facebook from the three largest drugstore chains in the United States was collected using APIs, RSS, HTML parsing, and manual copying.	Case study. Concept maps and sentiment analysis.	By comparing the social media use by three drugstore chains and their customer engagement trends, the study provides recommendations to help firms develop social media competitive strategies.
[14]	To propose a framework to illustrate how business decision-making can be derived from big social media data.	Retailing	User-generated content on Twitter from five large companies in the retail industry was collected using API.	Case study. Sentiment analysis, concept map, and theme analysis	The study provides practical guidance for valuable knowledge and business intelligence extraction from big social media data.
[20]	To explore how companies can learn from customer tweets to improve their online retail service.	Retailing	User-generated content on Twitter from five leading UK online retailers was collected.	Topic modelling, social network analysis, and sentiment analysis.	The study provides a novel framework to transform social media data into useful knowledge and improve online retailing service.
[18]	To propose a two-stage framework for analyzing social media content.	Tourism	User-generated content on Yelp from tourism brands was collected using a web crawler.	Case study. Sentiment analysis and machine learning classifier.	The study contributes to tourism and big data literature by developing a novel framework for extracting knowledge from social networks data and testing its capacity.
[21]	To illustrate how business competitive analysis can be used to detect promoted posts on social media.	Multiple industries	Data comprises of both brand posts and customers’ reactions was collected from Facebook using graph API.	Logistic regression, random forests, and extreme gradient boosting.	The study illustrates how social media data can be transformed into knowledge through models.
[13]	To predict start-up firms’ social media engagement level.	Multiple industries	Start-up firms’ twitter accounts information were collected using Twitter API.	Sentiment analysis, Machine learning models	The study addresses the problem of lacking methodology among start-up firms in predicting their social media marketing efforts.
[19]	To understand the most important features of active social media engagement and the impact of customer engagement rate on sales of the new product.	Hospitality	Social media posts on Facebook, Twitter, and Instagram from hospitality brand were collected.	Themes analysis, sentiment analysis, and regression model.	By illustrating the customer engagement rate of the brands, the study provides empirical evidence that highly engaged social media posts drive firm performance through increased sales.
[2]	To develop a social media analytic framework for improving operations and service management.	Retailing	User-generated content on Twitter from the three largest retail pharmacy organizations in the UK was collected using API.	Case study. Topic modelling, sentiment analysis, and concept maps.	The study provides insights into the use of social media data for developing social media strategies as well as improving operations and service quality.
Our study	To describe how social media competitive intelligence can be used for customer engagement prediction and decision-making.	Catering	Content generated by the leading catering brands in the United States in Twitter was collected using Twitter API.	Sector-based analysis. Sentiment analysis, topic modeling, machine learning based classifiers.	The study highlights the impact of external environment on the nature of data and the importance of competitive intelligence in customer engagement prediction and decision support.

Most studies focus on describing how to conduct a competitive analysis and convert data into actionable knowledge for decision-makers [14–16, 31]. Few studies discuss the specific benefits empowered by competitive intelligence, including service [2, 20], customer experiences [17], sales improvement [19], promoted post detection [21], and firms’ engagement level prediction [13]. Retailing is the most investigated industry. User-generated content (UGC) has been extensively investigated and has been collected mainly using the Application Programming Interface (API) of the Twitter Developer Platform (The term ’Twitter’ is used to refer to the platform now known as ’X,’ following its rebranding in 2023. This study captures a historical snapshot of the platform during the time it was still referred to as ’Twitter.’ Since Elon Musk’s acquisition and rebranding of the service, several changes have taken place that may impact the current relevance of findings derived from data collected during this earlier period. This is acknowledged in the limitations of the study, particularly regarding the challenges with the use of the X API for future research) [2, 14–16, 19, 20]. Sentiment analysis is the most commonly used text mining method to extract knowledge from unstructured data [13–16, 20], followed by topic/themes analysis [2, 14, 15, 19, 20, 31], concept maps [2, 17], and social network analysis [20]. The case study is the most used approach by researchers to demonstrate the implications of their proposed frameworks [2, 14, 15, 17, 18, 31]. The findings of existing studies reveal that social media competitive intelligence frameworks are effective in converting social media data into actionable knowledge, which can be used to enhance firms’ competitiveness in the market.

Nevertheless, we did not find a framework that incorporated the impact of the external environment on brand topics and the prediction of customer engagement. Firms’ marketing decisions are influenced by environmental uncertainty [5]. Researchers have found that topic patterns of CEOs in social media have changed after the breakout of the COVID-19 pandemic [22]. However, researchers have not revealed how environmental uncertainty affects topic patterns in FGC from brand-hosted social media accounts. The content from brand-hosted social media accounts is valuable because it affects customers’ satisfaction, brand gratitude, and customer engagement [32].

Below we propose a new framework in which the influence of the external environment on brand topics is highlighted and the phase to predict customer engagement is incorporated.

## 3. The proposed social media competitive intelligence framework

**Fig 1:**
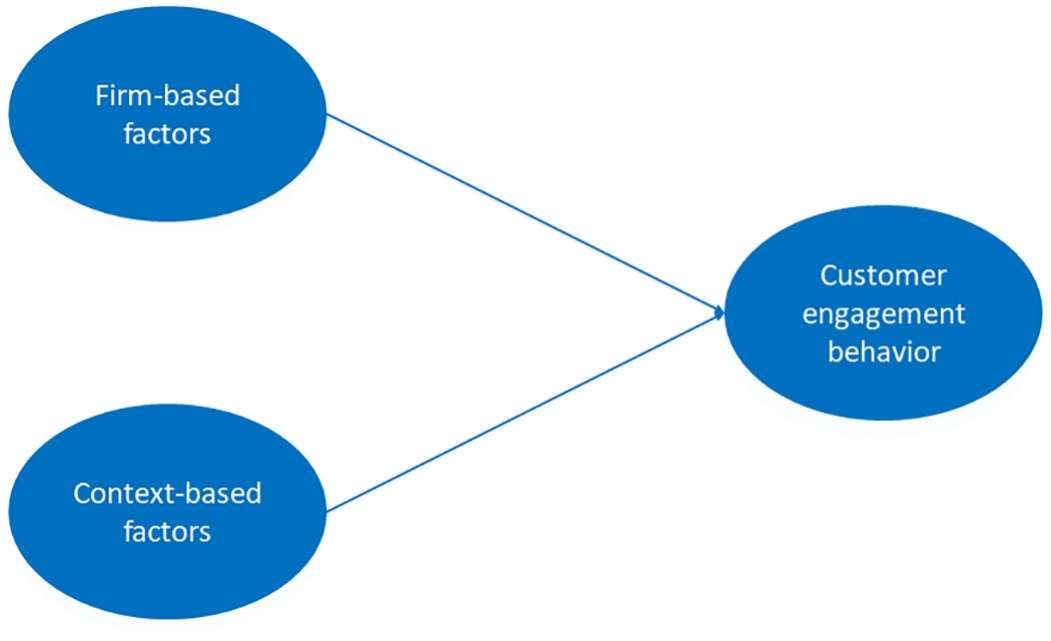
Conceptual social media competitive intelligence framework.

Our proposed framework (Fig 1) integrates the relationships between firm-based factors, environmental-based factors, and customer engagement. Drawing on [9], firm- and context-based factors serve as antecedents that jointly affect customer engagement.

Firm-based factors such as brand characteristics, firm processes, and marketing strategies significantly influence customer engagement behaviors [9]. These factors encompass the brand’s reputation, equity, and ability to engage customers through various platforms and processes for collecting and utilizing customer data, as well as the overall strategies to enhance customer satisfaction, trust, and engagement.

Environmental-based factors, including political, legal, economic, social, and technological environments, influence customer [9]. These factors affect the way customers interact with brands and influence the content and patterns of engagement on social media. For instance, the COVID-19 pandemic has had a substantial impact on customer behavior. Variables such as the number of COVID-19 infections and death tolls can drastically change how customers engage with brands. Regulations and policies can either facilitate or restrict information flow and customer interactions, while economic conditions and social trends, shaped by the pandemic, influence customer behaviors and brand interactions.

The external environment also affects data from competitors. Changes in the political, economic, social, and technological landscape influence the strategies and activities of competitors, which in turn affect their social media content and engagement metrics. For example, during the COVID-19 pandemic, competitors might change their marketing strategies, leading to variations in the timing, sentiment, and topics of their social media posts. These environmental factors create a dynamic interplay between competitive actions and customer engagement, necessitating a comprehensive framework to capture and analyze these interactions effectively.

**Fig 2:**
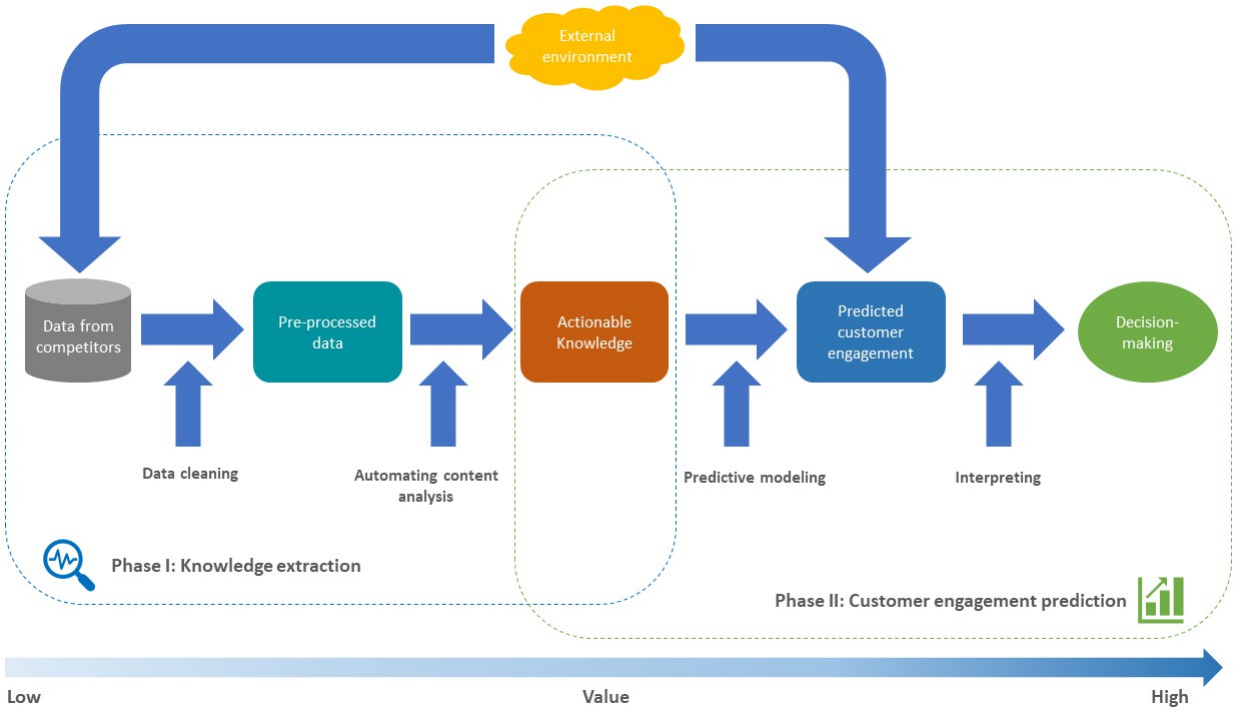
Operational social media competitive intelligence framework.

While the theoretical relationship between firm-based factors, environmental-based factors, and customer engagement is well-established, practitioners need a more structured approach to effectively apply these concepts. To measure, track, understand, and interpret the causes and consequences of marketplace behavior, researchers and managers require a comprehensive framework [33]. Therefore, we propose a specific social media competitive intelligence framework (Fig 2) that builds on the general concepts discussed above. This framework consists of two main phases: converting firm-generated content in social media into actionable knowledge and using this knowledge to predict customer engagement volume on social media.

The first phase starts with access to social media data. After the data collection, there will be two categories of data: structured data and unstructured data. Structured data, such as the number of likes, retweets, and comments, can be accessed and processed directly into a more advanced model [34]. In contrast, unstructured data, such as textual content, must be converted into a structured format [34]. Then, data cleaning must be conducted to process unstructured textual data, including lower-case letter transformation, punctuation, digit removal, stop word removal, and stemming/lemmatization [35].

Once cleaned data are ready to be processed, different natural language processing (NLP) techniques can be used to extract the knowledge from unstructured social media data, including topic modeling and sentiment analysis [36]. In our framework, we applied Latent Dirichlet Allocation (LDA), an unsupervised algorithm [37] for topic extracting. As for sentiment analysis, we suggest using supervised machine-learning approaches, which outperform unsupervised algorithms in automated text classification tasks [38]. Specifically, we recommend using pretrained supervised learning models in major cloud computing services (e.g., Amazon AWS, Google Cloud, and Microsoft Azure) to maximize the working efficiency, especially when the corpus to be analyzed contains a large number of documents. Previous findings suggest that cloud computing services significantly mitigate a series of informational and marketing barriers [39].

The second phase involves using the intelligence gained to predict customer engagement on social media, now also considering the influence of external environmental factors such as market trends, economic conditions, and global events like the COVID-19 pandemic. This analysis is crucial as these factors can significantly alter customer engagement patterns and competitor strategies. Predictive models are specified using structured features (i.e., sentiment and topic labels) and original structured data (e.g., published time, day, and media type) to forecast customer engagement, factoring in the external environmental influences (e.g., number of covid infections and deaths).

This two-phase framework effectively synthesizes data collection and analysis with predictive modeling. The first phase focuses on converting both structured and unstructured social media data into actionable knowledge through advanced NLP techniques and data cleaning processes. The second phase leverages this knowledge to develop predictive models that can accurately forecast customer engagement on social media. This comprehensive approach enables a deeper understanding of the dynamic social media landscape and aids in developing more responsive and effective social media strategies.

## 4. Implementation of the framework: An analysis of the leading American fast-food brands

Unlike previous social media competitive intelligence studies that have typically relied on case studies [15, 18], we employed a sector-based analysis to demonstrate how to implement our social media competitive intelligence framework. The catering industry was chosen as it was one of the sectors most affected by the COVID-19 pandemic [40–42], providing a valuable context for assessing shifts in brand communication and customer engagement. American brands were selected because the United States was among the countries most severely impacted by the pandemic, with high infection and death rates. While this study focuses on the American catering industry, the framework is not limited to this context and can be adapted for use in other industries, such as retail, healthcare, or entertainment, where social media plays a crucial role in customer interaction. Moreover, the approach is flexible enough to be applied in different geographical regions, allowing future research to explore how factors such as cultural differences or market-specific dynamics influence competitive intelligence outcomes.

In line with the proposed framework, the empirical research process was divided into two phases. In the first phase, we split the dataset into "Pre-pandemic" and "Pandemic" periods to extract sentiment and topic labels from the catering brands. This separation allowed us to capture and analyze the shifts in social media discussions and customer sentiments before and during the pandemic, providing a clear view of how the external environment influenced brand communication strategies.

In the second phase, we used the entire dataset without splitting it to predict customer engagement on social media. By not splitting the dataset in this phase, we aimed to leverage the complete range of data available to train and evaluate the predictive models. This approach ensured that the models were catered by the full spectrum of customer engagement behaviors, thereby enhancing their predictive accuracy and robustness. The performance of the predictive models was then evaluated based on their ability to accurately forecast customer engagement.

### 4.1. Phase one: Extraction of social media competitive intelligence

#### Methodology

From the ranking of the 25 most valuable restaurant brands in the world [43], we manually examined and selected the corresponding official brand accounts on Twitter using two criteria. First, the brand account should have been verified on Twitter. Second, the brand should operate its official Twitter account in the United States market. As Haidilao and Wetherspoons did not have official accounts in the United States, they were screened out. We utilized Twitter API V2 to collect tweets generated by the 23 selected food brands from March 1, 2019, to February 28, 2021. This period was chosen specifically to encompass both pre-pandemic and pandemic phases, with March 11, 2020, serving as the demarcation point when the World Health Organization officially declared the COVID-19 pandemic [44]. The end date of February 28, 2021, was selected to capture a full year’s worth of social media activity during the pandemic, allowing us to observe how brands adapted their communication strategies throughout this ongoing global disruption. By covering a two-year period, including both the pre-pandemic and pandemic phases, this timeframe provides sufficient data to analyze shifts in customer engagement and brand behavior. In total, we collected 9,733 tweets, excluding 666 retweets and 361 non-English tweets, leaving 8,721 original tweets in the final dataset.

For topic modeling, we utilized the LDA algorithm [37] to explore the textual data. LDA is a widely recognized and effective unsupervised machine learning technique for discovering hidden topics within large text corpora. It works by assuming that documents contain multiple topics and allocates words to these topics based on probability distributions [37]. LDA was chosen due to its interpretability, scalability, and established use in similar social media analyses, where uncovering underlying themes in unstructured text is essential. While newer methods such as BERTopic [45] or Top2Vec [46] are available, LDA’s simplicity and effectiveness in producing meaningful topics from short-form text like tweets make it a well-suited choice for this study.

For sentiment analysis, we employed Amazon Comprehend, a cloud-based machine learning service. Amazon Comprehend offers pre-trained models that continuously improve through retraining on large, diverse datasets, making it highly effective for processing sentiment at scale. Amazon Comprehend offers pre-trained models optimized for processing high volumes of text, making it particularly useful for social media datasets [47]. It continuously updates its models, ensuring high accuracy across diverse data types. Alternatives like VADER [48] or TextBlob [49] are frequently used for smaller datasets, but Amazon Comprehend’s scalability, ease of deployment, and high accuracy made it a preferable choice for our larger dataset of tweets.

#### Results

The basic information of the 23 catering brands is summarized in Table 2, including headquartered country, name of the Twitter Account in the United States, number of followers, number of followings, number of tweets (all time), number of tweets (in the period of 2019/03/01–2021/02/28), and percentage of tweets (in the period of 2019/03/01–2021/02/28).

**Table 2:** Basic information of the leading 23 catering brands (Data collected on September 10, 2021).

Ranking	Brand	Headquartered country	Twitter Account in United States	Number of followers	Number of following	Number of tweet (All time)	Number of tweet (2019/03/01–2021/02/28)	Percentage of tweets (2019/03/01–2021/02/28)
1	Starbucks	United States	@Starbucks	10923729	91123	249812	418	4.79%
2	McDonald’s	United States	@McDonalds	4296808	13543	758323	250	2.87%
3	KFC	United States	@kfc	1538619	11	48131	199	2.28%
4	Subway	United States	@SUBWAY	2293289	30022	107980	506	5.80%
5	Domino’s Pizza	United States	@dominos	1375448	536	447651	593	6.80%
6	Taco Bell	United States	@tacobell	1962103	74	795579	314	3.60%
7	Dunkin’	United States	@dunkindonuts	1230053	52786	104070	627	7.19%
8	Pizza Hut	United States	@pizzahut	1637011	77652	492046	497	5.70%
9	Tim Hortons	Canada	@TimHortonsUS	27852	2397	8479	218	2.50%
10	Wendy’s	United States	@Wendys	3809174	443	205704	479	5.49%
11	Burger King	United States	@BurgerKing	1953213	3827	45989	342	3.92%
12	Chipotle	United States	@ChipotleTweets	1056254	13	929069	480	5.50%
13	Chick-fil-A	United States	@ChickfilA	1132887	7270	200219	314	3.60%
14	Costa	United Kingdom	@costacoffeeus	328	33	93	61	0.70%
15	Jack In The Box	United States	@JackBox	118535	10129	40440	625	7.17%
16	Olive Garden	United States	@olivegarden	414806	18085	181071	265	3.04%
17	Texas Roadhouse	United States	@texasroadhouse	105512	28984	18588	148	1.70%
18	Papa John’s	United States	@PapaJohns	643840	5404	78090	662	7.59%
19	Chili’s	United States	@Chilis	402150	25593	414993	591	6.78%
20	Jollibee	Philippines	@JollibeeUSA	1017	0	116	0	0.00%
21	Cheesecake Factory	United States	@Cheesecake	354222	13397	38544	550	6.31%
22	Cracker Barrel	United States	@CrackerBarrel	116682	309	12366	174	2.00%
23	Popeyes	United States	@Popeyes	245698	5325	62907	408	4.68%

S1 Fig in the online material shows the volume of tweets published by the 23 catering brands from March 1, 2019 to February 28, 2021, and the dotted line represents the outbreak of the COVID-19 pandemic. We can observe that firms’ reactions to the pandemic are different in social media. While the volume for some brands (e.g., Dunkin’ and Costa) increased after the outbreak, the volume for others (e.g., Starbucks and Tim Hortons) did not change notably before and during the pandemic. Regarding the valence of the tweets (see S2 Fig in the online material), tweets with neutral sentiments dominated before and after the COVID-19 outbreak, followed by those with positive, negative, and mixed sentiments.

**Fig 3:**
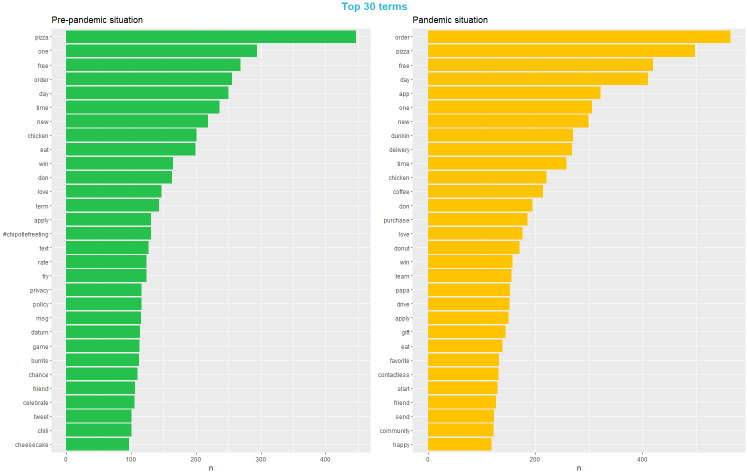
Top terms before and during the pandemic.

Following the best practices for conducting the LDA model [50], we preprocessed the unstructured textual data, removing uniform resource locators (URLs), digits, and special characters (except “#”, which refers to topic; and “@” which refers to mentions of another person or entity on Twitter). Then, we tokenized the documents in the corpus and converted all the words into the lower case before lemmatizing them. We used lemmatization instead of stemming because lemmas are more interpretable than stems [50]. Moreover, we removed the stop words (e.g. “a”, “the”, “is”, and “are”), because these words usually do not have analytical values in LDA modeling [35]. We listed the top 30 terms before and during the pandemic (Fig 3). The results show that the most frequently used words before and during the pandemic were different. Apart from the commonly appeared terms in the pre-pandemic situation like “order” or “pizza” during the pandemic, the catering brands were prone to use the terms in line with the marketing strategies used such as “app”, “delivery”, and “contactless.”

**Fig 4:**
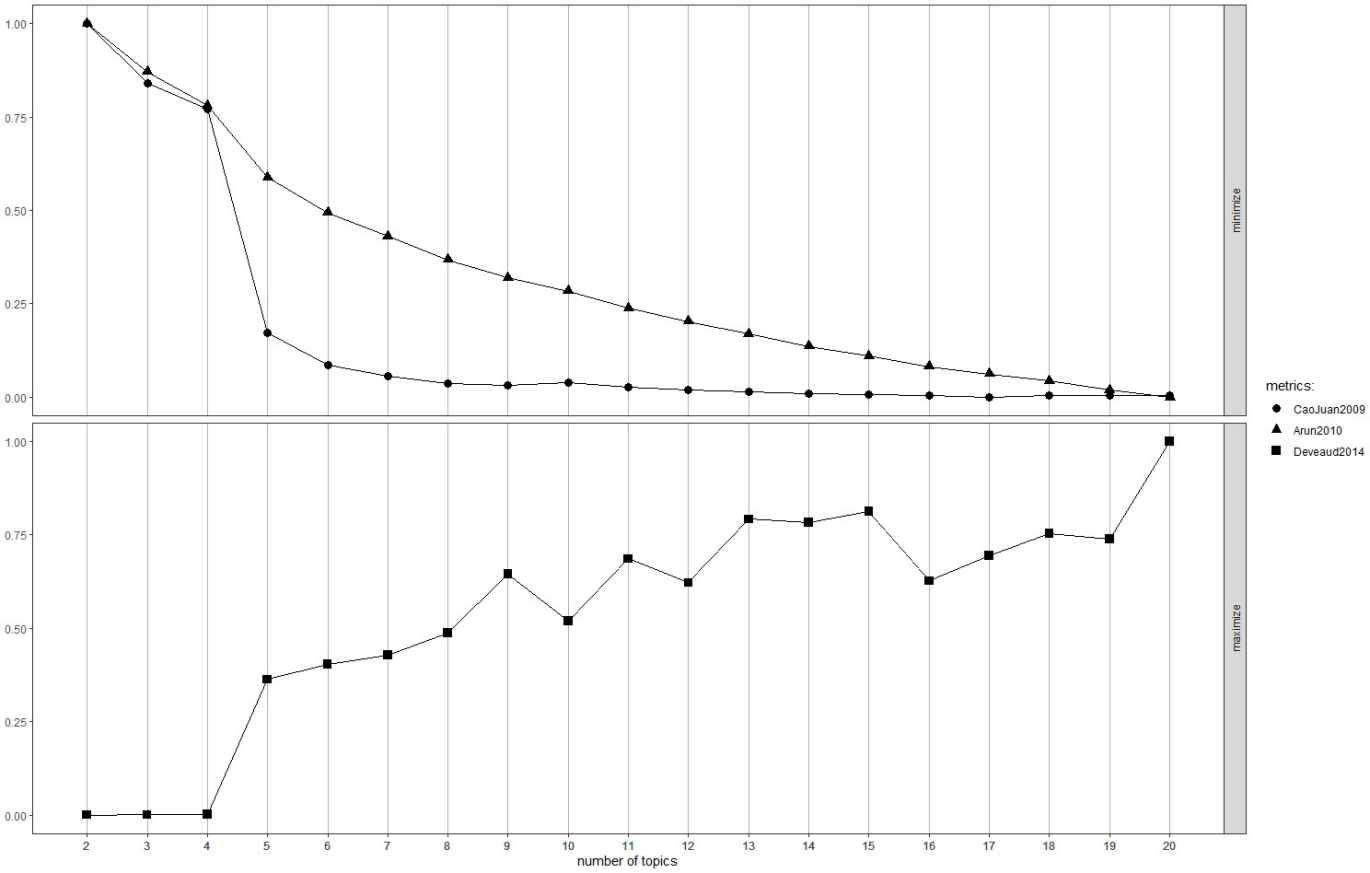
LDA model tuning for the "Pre-pandemic" subset.

**Fig 5:**
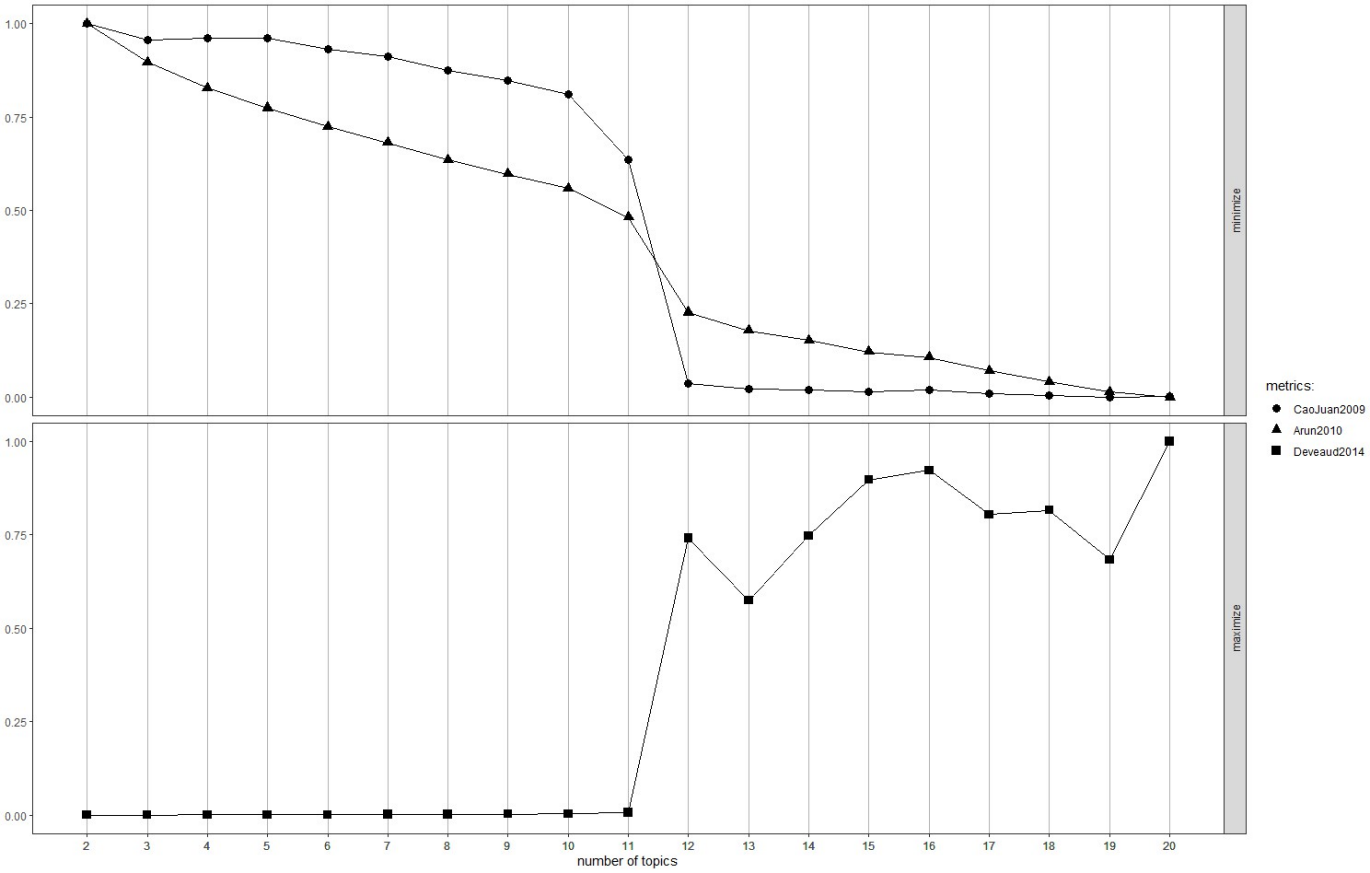
LDA model tuning for the "Pandemic" subset.

When specifying an LDA model, the number of topics, k, must be defined *a priori* [50]. We used the *FindTopicsNumber* function of the *ldatuning* package to automate this process. We tested 19 LDA models specifying k values ranging from 2 to 20, and evaluated their performances using the Cao Juan metric [51], Arun metric [52], and Deveaud metric [53]. An LDA model with lower Arun and Cao Juan metrics and higher Deveaud metrics suggests a better solution. The results of the model tuning are shown in Figs 4 and 5. On the one hand, in the LDA models with data from the “Pre-pandemic” subset (see Fig 4), an explicit elbow appears when the k value is equal to 5. On the other hand, in the LDA models with data from the “Pandemic” (see Fig 5), the explicit elbow can be found when the k value equals 12. These results indicate that 5 and 12 are the optimal k values for the "Pre-pandemic" and "Pandemic" subsets, respectively, as these values offered the most suitable model performance without adding unnecessary complexity. Beyond these points, further increases in the number of topics yielded diminishing returns in terms of model quality.

**Fig 6:**
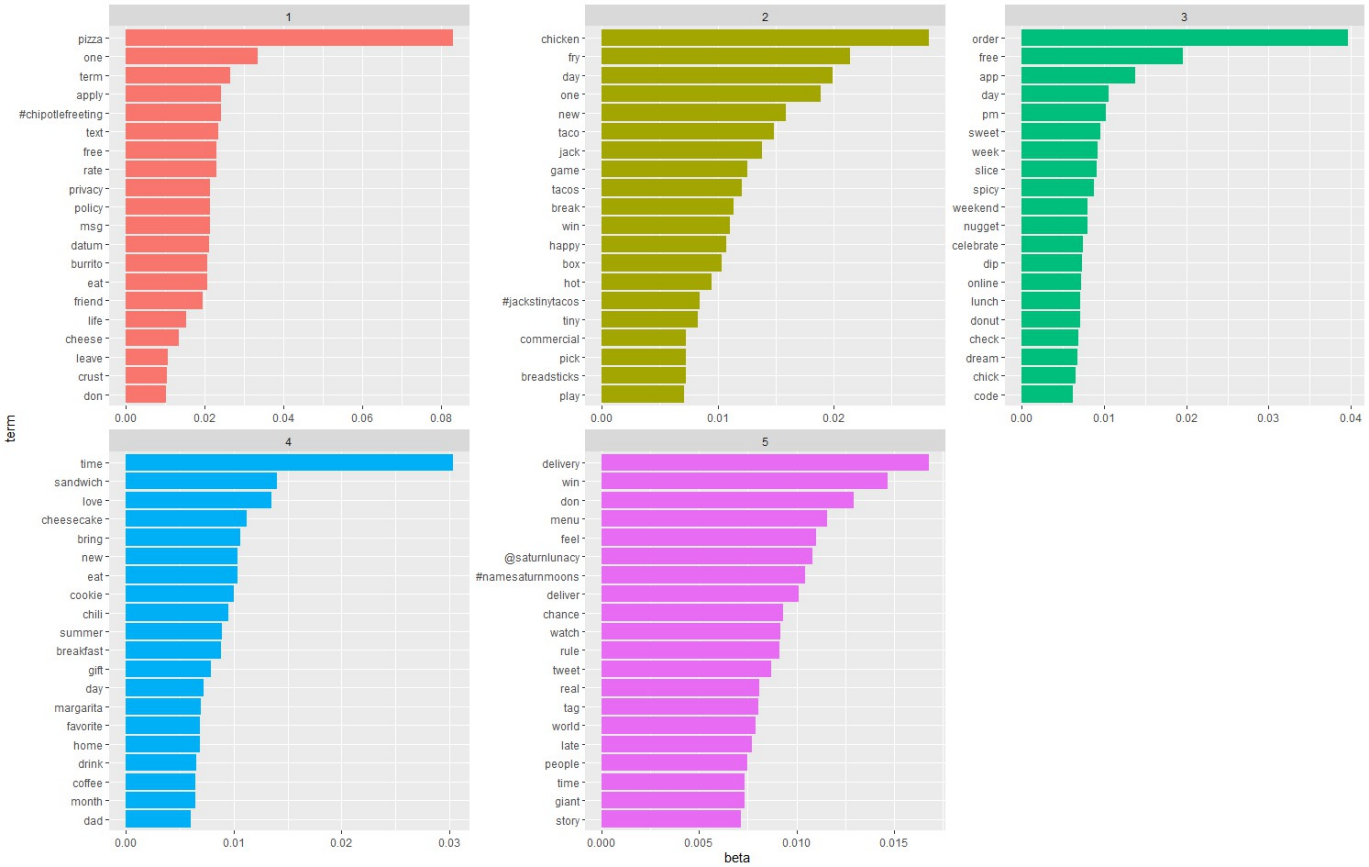
Visualisation of beta values for LDA topics in the "Pre-pandemic" subset.

**Fig 7:**
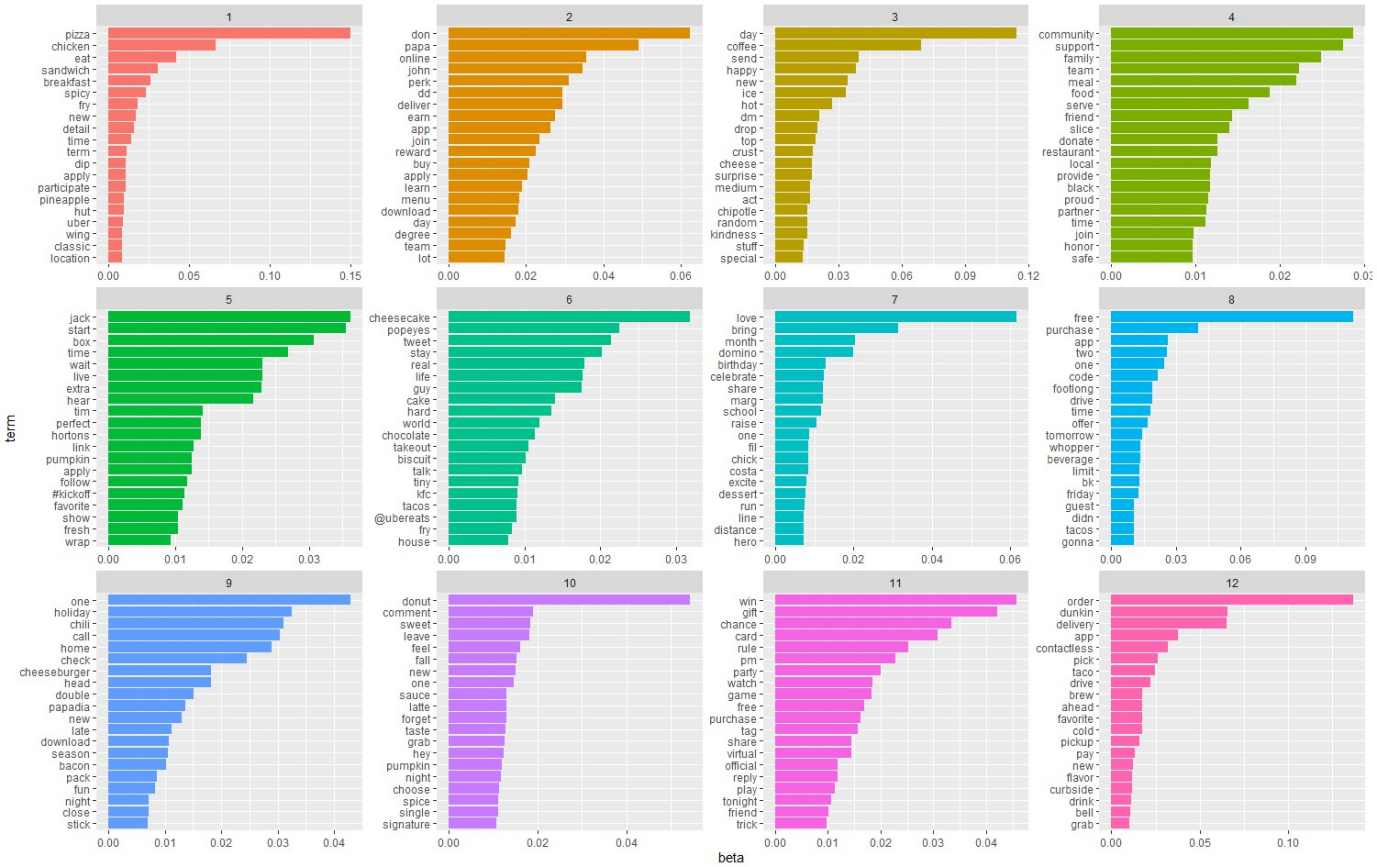
Visualisation of beta values for LDA topics in the "Pandemic" subset.

After confirming the key values, we used variational expectation maximization (VEM) sampling for LDA modeling. We used VEM instead of Gibbs sampling because previous empirical evidence suggests that VEM performs better in corpuses with a large number of documents [54]. After applying the LDA algorithm for the “Pre-pandemic” and “Pandemic” subsets, we obtained the beta values for the terms in each topic. The beta value reveals the probability that a term belongs to a topic [55]. Figs 6 and 7 show the visualizations of beta values from both subsets.

Using the visualizations of the beta values to interpret the results of LDA is not an easy task [55]. Thus, we also considered documents with high gamma values (gamma > 0.7) when interpreting the results. The gamma value reveals the probability that a document belongs to a specific topic [55]. Later, we labeled the topics suggested by the LDA algorithm considering the terms with high beta values and documents with high gamma values. We found that the documents in the “Pre-pandemic” subset pertain to the following topics: “1-Food and lifestyle”, “2-Promotion”, “3-Food ordering”, “4-Food time”, and “5-Food delivery”. The documents in the “Pandemic” subset pertain to the following topics: “1-Food time”, “2-Coupons and offers”, “3-Theme day for foods”, “4-Social responsibility”, “5-Food and lifestyle”, “6-Brand specialty”, “7-Warmth conveying”, “8-Calls to purchase”, “9-News sharing”, “10-Sense of taste”, “11-Event promotion”, and “12-Contactless ordering and delivery.” We summarize the labeled topics with descriptions and examples (see S1 Table in the online material).

**Fig 8:**
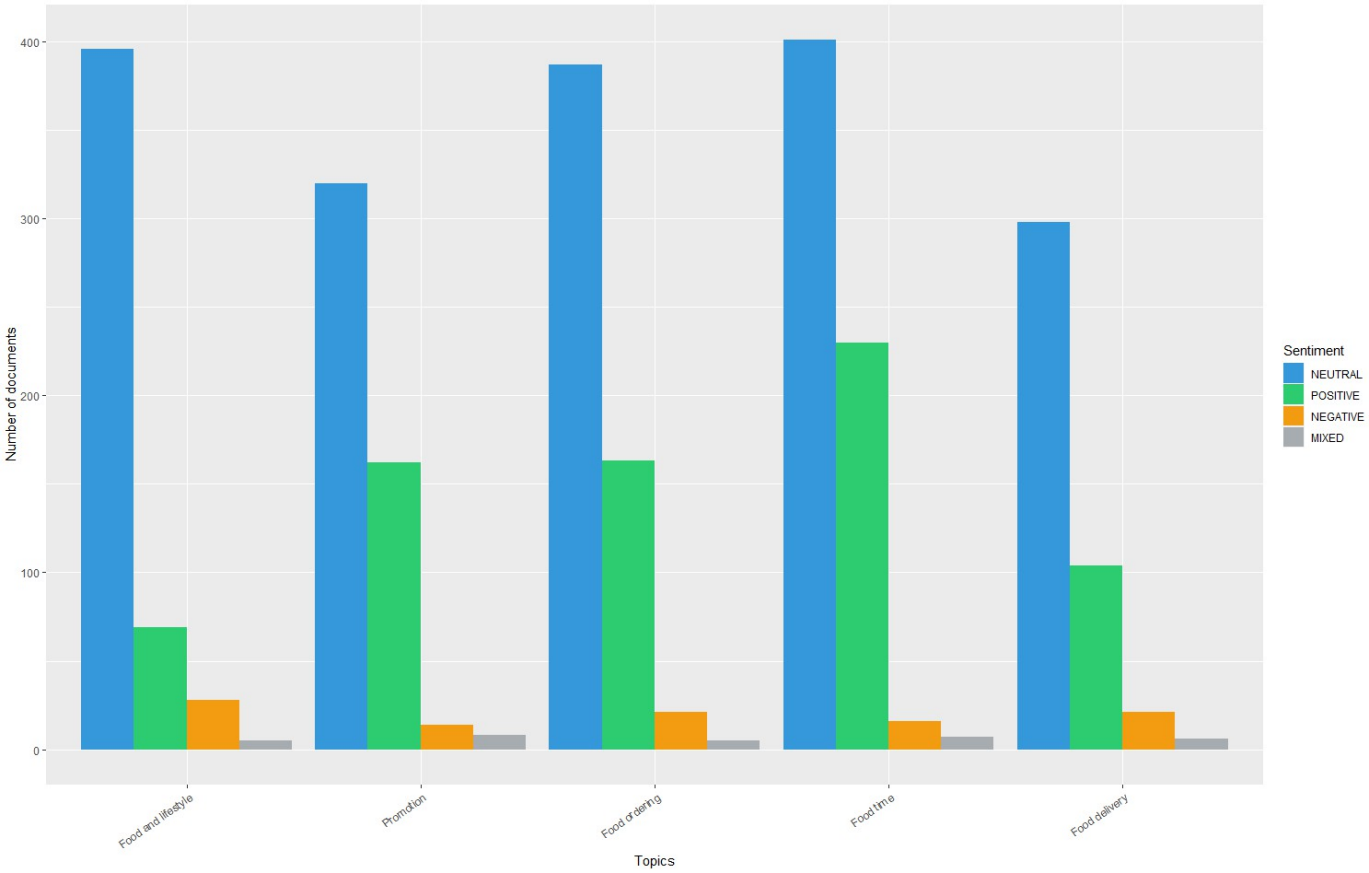
Sentiment analysis of topics in the "Pre-pandemic" subset.

**Fig 9:**
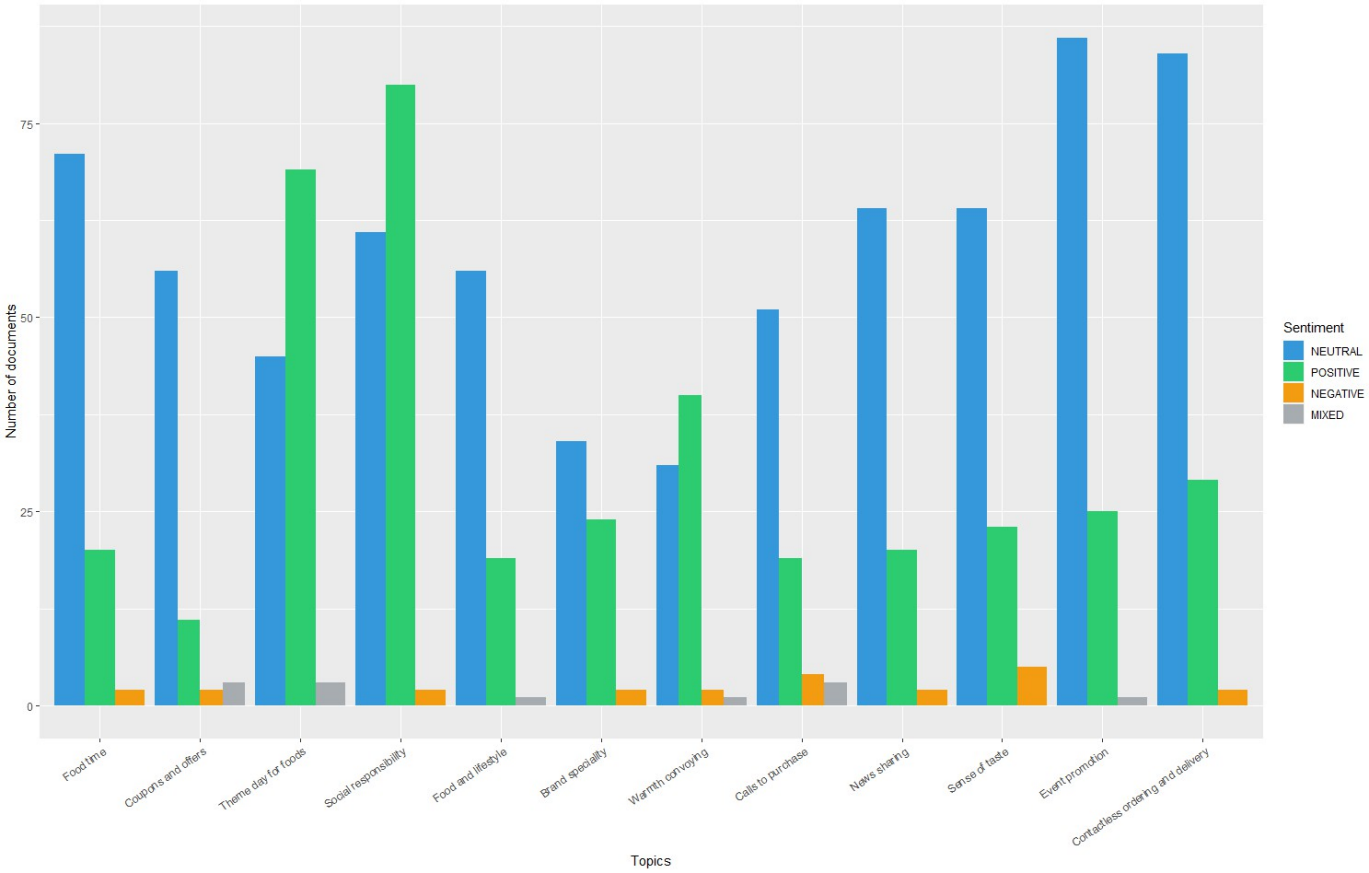
Sentiment analysis of topics in the "Pandemic" subset.

Figs 8 and 9 show the sentiments in each topic before and during the pandemic. Catering brands tend to use a neutral tongue when posting tweets. However, they tend to express positive emotions for three topics during the pandemic, including “Theme day for foods”, “Social responsibility”, and “Warmth conveying”. Moreover, they rarely use negative or mixed tongues in all topics, whether before or during the pandemic.

### 4.2. Phase two: Prediction of customer engagement in social media

#### Methodology

In this phase, we used structured textual data along with other originally structured data to predict customer engagement in social media. We illustrate the predictive function of the social media competitive intelligence framework using likes, replies, and retweets as the outcome variables. These three dimensions of customer engagement were selected because they each represent different levels of user interaction and influence on social media [28]. Likes indicates a basic level of appreciation, replies involve direct interaction, and retweets demonstrates content rebroadcasting, which can significantly extend the message’s reach at no additional cost to firms. Through these engagement behaviors, marketing practitioners can reach potential customers who are not actively following the brand account of their firm [56].

One of the challenges faced by firms when conducting social media competitive analytics is determining how their performance stacks against their key competitors’ performance [16]. Therefore, setting a benchmark is important in social media competitive intelligence frameworks [16]. We set the benchmarks for engagement volumes to 194 for likes, 24 for replies, and 25 for retweets, as these were the median values of the sampled brands in the catering industry. This means that if a firm’s messages on Twitter consistently exceed these benchmark values, the firm gains a competitive advantage over some of its competitors. Therefore, we trained the classification models to select the models that most accurately predicted users’ engagement behaviors across all three dimensions.

Based on the literature, we selected nine independent variables as the predictors of users’ retweet behavior: Food type [57], Published time and day of tweet [58–60], Media type [60–63], Number of mentions [64], Number of hash tags [65], Number of Links [60–63], Sentiment [62], Topic [56, 66], COVID-19 infected numbers [7] and COVID-19 death toll [7] (see Table 3).

**Table 3:** Description of the variables used in the predictive modeling.

Variable	Description	Reference
Food type	The rands are divided into six class according to the subsector that they pertain in the catering industry: "Burger/Sandwich", "Casual dining", "Coffee/Dessert", "Fried chicken", "Pizza/Italian food", or "Taco/Mexican food"	[57]
Published time of tweet	The published time of the tweets, according to the Eastern Standard Time (EST): Morning (from 6:00 to 11:59), Afternoon (from 11:59 to 19:00), or Night (from 19:00 to 5:59)	[58–60]
Published day of tweet	The published day of the tweets, according to the Eastern Standard Time (EST): "Monday", "Tuesday", "Wednesday", "Thursday", "Friday", "Saturday", or "Sunday"	[57–60]
Media type	The type of the media in a tweet: "No media", "Image", "Gif", or "Video"	[60–63]
Number of mentions	The count of mentions (using the "@" symbol) in the tweet.	[64]
Number of hash Tags	The count of hashtags (using the "#" symbol) used in the tweet.	[65]
Number of links	The count of links (URLs) included in the tweet.	[60–63]
Sentiment	The sentiment in the tweet: "Neutral", "Positive", "Negative", or "Mixed"	[62]
Topic	The topic extracted from the tweet.	[56, 66]
COVID-19 infected numbers	The number of reported COVID-19 cases in United States on the day of the tweet, as a reflection of the pandemic’s severity and public concern.	[7]
COVID-19 death tolls	The number of reported COVID-19 related deaths in United States on the day of the tweet, as a reflection of the pandemic’s severity and public concern.	[7]

We used six state-of-the-art machine learning algorithms to specify the classification model including the: support vector machine (SVM) with radial basis kernel [67], CART tree [68], C5.0 tree (an improved version of the original C4.5 algorithm [69], random forest [70], bagged CART [71], and gradient boosting [72]. The aforementioned algorithms were selected as candidates for the final classifier because they are the most used machine learning algorithms to conduct the classification task [73].

We included documents with high gamma values (gamma > 0.7) from topic modeling (Phase 1) in the predictive model. Gamma values represent the probability that a document belongs to a particular topic, with higher values indicating a stronger association with that topic. This threshold ensures that only documents strongly associated with specific topics are included, enhancing the robustness and relevance of the predictive model. After applying this criterion, there are now 3378 observations in the dataset, providing a focused sample for further analysis.

When applying machine learning-based predictive models, there are several challenges. The first challenge is the problem of overfitting. Overfitting occurs when the model learns the characteristics of each sample’s unique noise instead of the general pattern in the data [73]. To reduce model overfitting, as suggested in the literature [74], we used *k*-fold cross-validation. Specifically, we used repeated 10-fold cross-validation, as this method is ideal for datasets with a limited sample size [73]. Another challenge of applying a machine learning approach is that many algorithms have important parameters that cannot be directly estimated from the data. This type of model parameter is referred to as a tuning parameter because there is no analytical formula available to calculate the value *a priori* [73].

To ensure the robustness of the six machine learning algorithms, we followed a structured resampling and tuning process, as outlined by [73]. First, we defined a set of candidate values for key hyperparameters, such as the number of trees in Random Forest, the complexity parameter in Decision Trees, and the learning rate in boosting algorithms (see S2 Table for the specific tuning parameters used for each algorithm). Next, we applied repeated 10-fold cross-validation, where the dataset was divided into 10 equal-sized folds. Each model was trained on 9 folds and validated on the remaining fold. This process was repeated 5 times, allowing each fold to serve as the validation set once, which helped to minimize bias and ensure stable performance across different subsets of the data. We used 80% of the dataset for training and reserved the remaining 20% for testing the final model. To address potential imbalances in the dependent variable, we applied stratified random sampling within each fold, ensuring that the proportion of classes (e.g., engagement levels) remained consistent across both training and validation sets. During the cross-validation phase, we evaluated the models based on accuracy and Kappa to assess performance and guide hyperparameter tuning. Once the optimal hyperparameters were selected based on the cross-validation results, we refitted each model using the entire training set. These models were then tested on the holdout set (20% of the data) to assess their generalization to unseen data. For the final evaluation, we used additional metrics such as sensitivity, specificity, positive predictive value (PPV), and negative predictive value (NPV), in addition to accuracy and Kappa, to provide a more comprehensive assessment of model performance.

Accuracy and Kappa were primarily used to assess overall model effectiveness, as they are widely recognized metrics for evaluating machine learning classifiers [75]. Given that we were addressing a two-level classification problem for each engagement dimension—“Low engagement” and “High engagement”—with thresholds set at 194 for likes, 24 for replies, and 25 for retweets, these metrics offered clear indicators of model accuracy. However, to gain a more nuanced understanding of model performance, we included sensitivity, specificity, PPV, and NPV. These additional metrics provided diagnostic insights, particularly for evaluating how well the models distinguished between low and high engagement classes [73]. For each engagement dimension, the high engagement class was treated as the positive event, as it represented the primary outcome we aimed to predict with the highest accuracy.

For the software environment, we used the caret package in the RStudio environment [76] to train and test the classifiers.

#### Results

S3 and S4 Tables present the descriptive statistics for the categorical and continuous variables used in the predictive modeling.

Table 4 presents the performance metrics of various predictive models used for classifying customer engagement (likes, replies, and retweets).

**Table 4:** Metrics of the predictive models.

Dependent_variable	Model	Cross-validation		Testing set					
		Accuracy	Kappa	Accuracy	Kappa	Sensitivity	Specificity	PPV	NPV
Like (Likes < 194 / Likes > = 194)	SVM	0.730	0.460	0.703	0.408	0.739	0.670	0.676	0.734
	CART	0.718	0.437	0.707	0.416	0.739	0.678	0.681	0.736
	C50	0.747	0.493	0.735	0.469	0.717	0.752	0.729	0.741
	Random forest	0.747	0.494	0.743	0.487	0.777	0.711	0.715	0.774
	Bagged CART	0.736	0.471	0.723	0.445	0.706	0.739	0.716	0.730
	Gradient boosting	0.728	0.456	0.709	0.417	0.717	0.701	0.690	0.727
Reply (Reply < 24 / Reply > = 24)	SVM	0.740	0.480	0.747	0.494	0.747	0.747	0.733	0.760
	CART	0.723	0.445	0.683	0.367	0.698	0.670	0.663	0.704
	C50	0.755	0.508	0.759	0.517	0.750	0.767	0.750	0.767
	Random forest	0.751	0.501	0.744	0.488	0.728	0.760	0.738	0.750
	Bagged CART	0.746	0.492	0.751	0.501	0.720	0.780	0.753	0.749
	Gradient boosting	0.722	0.443	0.732	0.465	0.731	0.734	0.719	0.745
Retweet (Retweets < 25 / Retweets > = 25)	SVM	0.698	0.397	0.703	0.407	0.749	0.659	0.683	0.728
	CART	0.681	0.363	0.665	0.330	0.674	0.656	0.658	0.672
	C50	0.719	0.437	0.709	0.417	0.735	0.682	0.694	0.724
	Random forest	0.724	0.448	0.722	0.444	0.767	0.677	0.700	0.748
	Bagged CART	0.716	0.433	0.713	0.425	0.738	0.688	0.699	0.728
	Gradient boosting	0.699	0.399	0.690	0.380	0.698	0.682	0.683	0.697

For predicting likes, the Random Forest model demonstrated the highest performance, with an accuracy of 0.743 and a Kappa of 0.487 on the testing set. This model also exhibited high sensitivity (0.777) and specificity (0.711). The C50 model also performed well, achieving an accuracy of 0.735 and a Kappa of 0.469.

In predicting replies, the C50 model showed the highest accuracy at 0.759 and a Kappa of 0.517 on the testing set, with balanced sensitivity (0.750) and specificity (0.767). The Random Forest model also performed strongly, with an accuracy of 0.744 and a Kappa of 0.488.

For retweets, the Random Forest model again demonstrated strong performance, with an accuracy of 0.722 and a Kappa of 0.444 on the testing set. It achieved high sensitivity (0.767) and reasonable specificity (0.677). The C50 model also showed notable performance, with an accuracy of 0.709 and a Kappa of 0.417.

In summary, the Random Forest and C50 models generally outperformed others in predicting customer engagement metrics, indicating their robustness for this application. The descriptive statistics provide valuable insights into the characteristics of the dataset, highlighting variations in tweet content, timing, sentiment, and engagement.

## 5. Discussion and implications

### 5.1. Discussion

Our study advances the foundational work of [18, 15] by introducing a new framework for social media competitive intelligence that highlights the impact of the external environment on user-generated content and emphasizes the value of gained knowledge for predicting customer engagement. By applying this framework to data from 23 catering brands in the United States, we demonstrated how intelligence derived from unstructured Twitter data can be effectively extracted and utilized to support marketing decisions. Our findings underscore the superior performance of ensemble methods, providing a nuanced understanding that complements and extends previous research, and illustrating the practical application of our framework in predicting social media engagement behaviors including likes, replies, and retweets. In phase 1 of the empirical study, we found that neutral sentiment predominated in the firm-generated content on Twitter, followed by positive, negative, and mixed sentiments. This finding is consistent with the results of previous research, in which four catering services of two retail chains were investigated [15]. Contrary to intuition, firms tend to publish content with neutral rather than positive sentiment in their commercial communication on social media. A possible explanation for this is that brands may wish to project an impartial and objective image to social media users, particularly during sensitive periods such as the COVID-19 pandemic. By adopting a neutral tone, firms can avoid the risk of alienating or polarizing certain segments of their audience, thus maintaining a broader appeal.

Through topic modeling, we identified five topics in the pre-pandemic situation data and twelve in the pandemic situation data. The empirical results indicate that catering brands were taking different strategies before and during the pandemic. Specifically, the results show that some topics exist in both the pre-pandemic and pandemic situations, including “Food and lifestyle” and “Food time.” Moreover, during the pandemic, firms created a new topic related to the “Sense of taste”. This is a type of marketing strategy called experiential customer engagement marketing initiative, which centers on intrinsically motivating customers by estimating heightened psychological and emotional connections to brands [77].

The topic “Promotion” in the pre-pandemic situation is further refined into six topics in the pandemic situation, which are “Coupons and offers”, “Event promotion”, “Calls to purchase”, “Theme day for foods”, “Brand specialty”, and “News sharing.” A possible explanation for this change is that firms respond to the economic effects of the pandemic on the business. During the pandemic, the survival of a business depends on firms’ ability to treat adverse situations as a turning point [78]. Our results reveal that firms were refining their marketing strategies to maintain their competitiveness. Additionally, the topics “Food ordering” and “Food delivery” are merged into one topic, “Contactless ordering and delivery.” We considered this shift to be related to customers’ behavioral changes. Due to the lockdown in the pandemic, the dine-in service of catering brands was limited [42]. Therefore, one of the effects of the pandemic on consumption is that “store comes home” [79]. Thus, during the pandemic, there was no need for catering brands to separate these two topics, because customers were ordering food from home, which tied up ordering and delivery. Also, the topic “Social responsibility” was found in the pandemic situation data, which revealed that firms paid more attention to social responsibility during the pandemic. The pandemic has started a new era in the relationship between corporate social responsibility and marketing [5]. Researchers suggest that the pandemic offers great opportunities for firms to actively engage in various social responsibility initiatives during the crisis [5] and our results indicate that firms are implementing this suggestion.

In phase 2 of our empirical study, we utilized competitive intelligence derived from unstructured social media data, including sentiments and topics, alongside structured data to predict customer engagement behaviors such as likes, replies, and retweets. Six machine learning algorithms were tested for their predictive capabilities.

The Random Forest model demonstrated superior performance across all engagement metrics. For likes, it achieved the highest accuracy (0.743) and Kappa (0.487), coupled with the highest sensitivity (0.777). Similar trends were observed for replies and retweets, where Random Forest maintained high accuracy and sensitivity, underscoring its versatility and effectiveness. The C50 model also performed commendably, particularly in predicting replies, where it achieved the highest testing accuracy (0.759) and Kappa (0.517). These findings highlight the robustness of Random Forest as an ensemble method in handling the complexities inherent in social media data. Moreover, the C50 model, while not an ensemble method, demonstrated strong predictive capabilities and interpretability, making it a valuable tool for specific predictive tasks.

Previous research has underscored the potential of machine learning models in predicting social media engagement. For instance, [13] demonstrated that complex models, including deep learning, achieved the highest accuracy for predicting customer engagement on social media. Our findings, however, indicate that ensemble methods, particularly Random Forest, offer superior performance. This observation aligns with previous evidence [21], in which researchers also found ensemble methods to be highly effective in social media analytics, thereby corroborating our results. Additionally, the accuracy of decision trees is not as high as that of Random Forest, which is in line with previous research [13]. However, the C50 model, with its balanced approach combining both high accuracy and interpretability, makes it a valuable tool for businesses that require transparent and explainable models.

While our study provides valuable insights into customer engagement and brand strategies through social media competitive intelligence, it is important to consider the ethical implications of utilizing social media data for research purposes. Social media platforms, such as Twitter, provide publicly available data, but users may not always be fully aware that their posts could be collected and analyzed by third parties for research or commercial purposes. This lack of explicit user consent raises concerns about privacy and data ownership. Although we strictly adhered to Twitter’s terms of service and used only publicly available information, researchers must recognize the ethical responsibility to ensure that individual privacy is respected, and that data is handled with care. Furthermore, the potential for profiling or exploitation of user-generated content by firms poses additional ethical concerns, as insights gained from social media data could be used to influence user behavior in ways that may not always align with their best interests. It is essential for future research to develop guidelines and protocols that safeguard user privacy and ensure transparency in data usage. This will help maintain public trust and protect individuals from unintended consequences of data-driven decision-making processes.

### 5.2. Implications for theory and practice

On the theoretical side, we contributed to the social media competitive intelligence literature by proposing a new conceptual framework. Although competitive intelligence generates important business value for firms [25], there is a gap between intelligence gaining and decision-making. We highlighted the influence of the external environment on brand topics and incorporated the predictive phase of customer engagement in the social media competitive intelligence framework. This adds to the literature by expanding the scope of the conceptual framework from merely knowledge extraction to customer engagement prediction.

On the practical side, we illustrated the implementation of the proposed social media competitive intelligence framework. The empirical results of the sector-based analysis reveal the specific sentiment and topic patterns of the main players in the American catering industry. These results are beneficial for marketing practitioners to analyze evolutionary trends in the catering industry before and during the pandemic and to predict customer engagement. The brand topic patterns in FGC have changed after the outbreak of COVID-19 pandemic: while some topics persist during the pandemic, some are replaced by others. This information can guide firms to make appropriate marketing decisions, especially when facing environmental uncertainties, as firms can continuously monitor how competitors adapt their marketing strategies to engage with customers in the changing environment. Besides, firms are recommended to incorporate the Random Forest algorithm into their social media competitive intelligence frameworks when the primary goal is to achieve the highest predictive accuracy. For businesses that also value model interpretability, the C50 model offers a balanced approach, ensuring both reliable predictions and ease of understanding. By selecting the appropriate algorithm based on their specific objectives, firms can enhance their ability to predict customer engagement and make more informed marketing decisions.

## 6. Limitations and future research

This research is not without limitations. First, in this study customer engagement in social media is only measured by the number of retweets. As customer engagement is a multidimensional concept [80], researchers can also focus on other dimensions and operationalize customer engagement in social media with other measures, such as the number of likes and comments [8].

Second, a notable limitation of this empirical study is the use of machine learning algorithms, which, unlike traditional statistical models, do not provide estimations of the coefficients for each independent variable. This makes it challenging to quantify the specific contribution of each predictor to the overall model. While machine learning models like Random Forest and C50 can offer high predictive accuracy and handle complex data patterns, their “black-box” nature means that they lack the transparency and interpretability of statistical models, where the impact of each independent variable can be clearly identified and understood through coefficient estimates. Future researchers are encouraged to explore hybrid approaches that combine the predictive power of machine learning algorithms with the interpretability of traditional statistical models. Techniques such as SHAP (SHapley Additive exPlanations) [81] or LIME (Local Interpretable Model-agnostic Explanations) [82] can be employed to provide insights into the contributions of individual predictors within machine learning models. Integrating these methods could offer a more comprehensive understanding of the factors driving social media engagement, balancing the need for both precision and interpretability.

Third, despite the strong predictive performance of models such as Random Forest and C5.0, there is a risk of overfitting, particularly with more complex models like Random Forest. Overfitting occurs when a model becomes overly tailored to the training data, capturing noise rather than general patterns, which can negatively impact its ability to generalize to new, unseen data. Although we employed strategies such as cross-validation and stratified sampling to mitigate overfitting, it remains a potential limitation, especially when dealing with smaller or imbalanced datasets. Future research could further explore techniques such as regularization or pruning to minimize overfitting, or experiment with simpler models that may offer better generalizability while still maintaining sufficient predictive power.

Fourth, in our empirical study, thirteen predictors were used to train and test the classifiers. While these predictors provided valuable insights, incorporating additional variables, such as UGC and geographical data, could potentially further enhance the model’s performance. Research has shown that user behavior on social media can vary based on cultural value orientations, demographic characteristics, and geographical location [83]. As our study focused on FGC without incorporating variables such as UGC or the geographical location of users engaging with the content, future studies are encouraged to explore these additional dimensions. Including UGC and accounting for geographical or cultural variations in engagement behavior could provide deeper insights and enhance the robustness of competitive intelligence frameworks in social media contexts.

Finally, it is important to note that the data used in this study were collected from ’Twitter’ (now rebranded as ’X’) during the period before Elon Musk’s acquisition and the platform’s subsequent changes in operations and user base. This study thus offers a historical snapshot of social media competitive intelligence frameworks during that earlier period. Future research may find that the engagement patterns and user behaviors observed here differ from current trends on ’X’, particularly due to the departure of certain businesses and users. Additionally, changes in the X API may present significant challenges for researchers seeking to collect and analyze data from the platform moving forward. Following the rebranding and leadership transition, access to the X API has become more restrictive, particularly with the introduction of paid access tiers and limitations on free access. These changes could result in reduced data availability for researchers, increased costs for accessing comprehensive datasets, and potential limitations on the types of data that can be retrieved (e.g., only retrieving limited historical data or reduced access to user engagement metrics). This evolving API structure may limit the scope of future research, necessitating alternative methods for data collection or analysis, such as relying on third-party tools or developing custom solutions to capture real-time data from the platform.

## Supporting information

S1 Fig Volume of tweets published by the catering brands from before and after the COVID-19 outbreak. (PNG)

S2 Fig Valence of tweets published by the catering brands before and after the COVID-19 outbreak. (PNG)

S1 Table Labeled topics with descriptions and examples. (DOCX)

S2 Table Parameters used in model tuning. (DOCX)

S3 Table Descriptive statistics of categorical variables used in predictive modeling. (DOCX)

S4 Table Descriptive statistics of continuous variables used in predictive modeling. (DOCX)
