# Supplementary figures and images for: A social media competitive intelligence framework for brand topic identification and customer engagement prediction
Source: PLoS One. 2024 Nov 25;19(11):e0313191. doi: 10.1371/journal.pone.0313191 (PMC11588230; doi:10.1371/journal.pone.0313191)

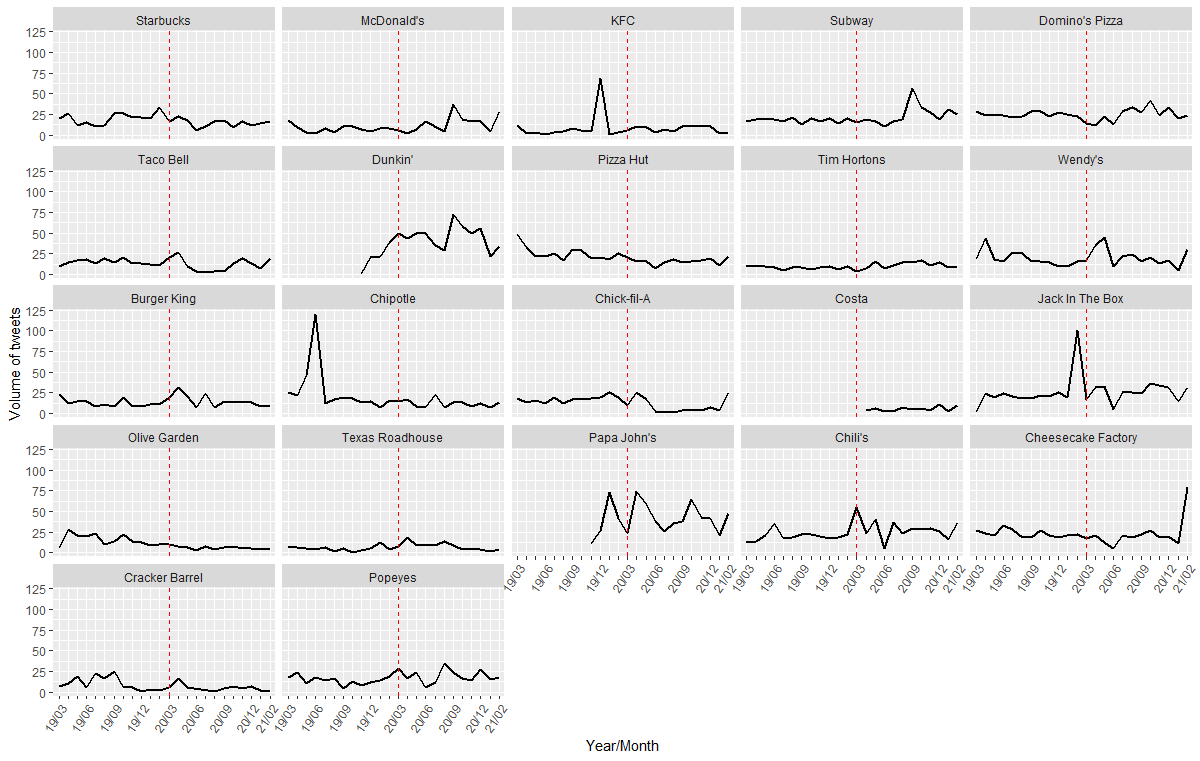

Supplement: S1 Fig — (PNG) [file pone.0313191.s001.png]

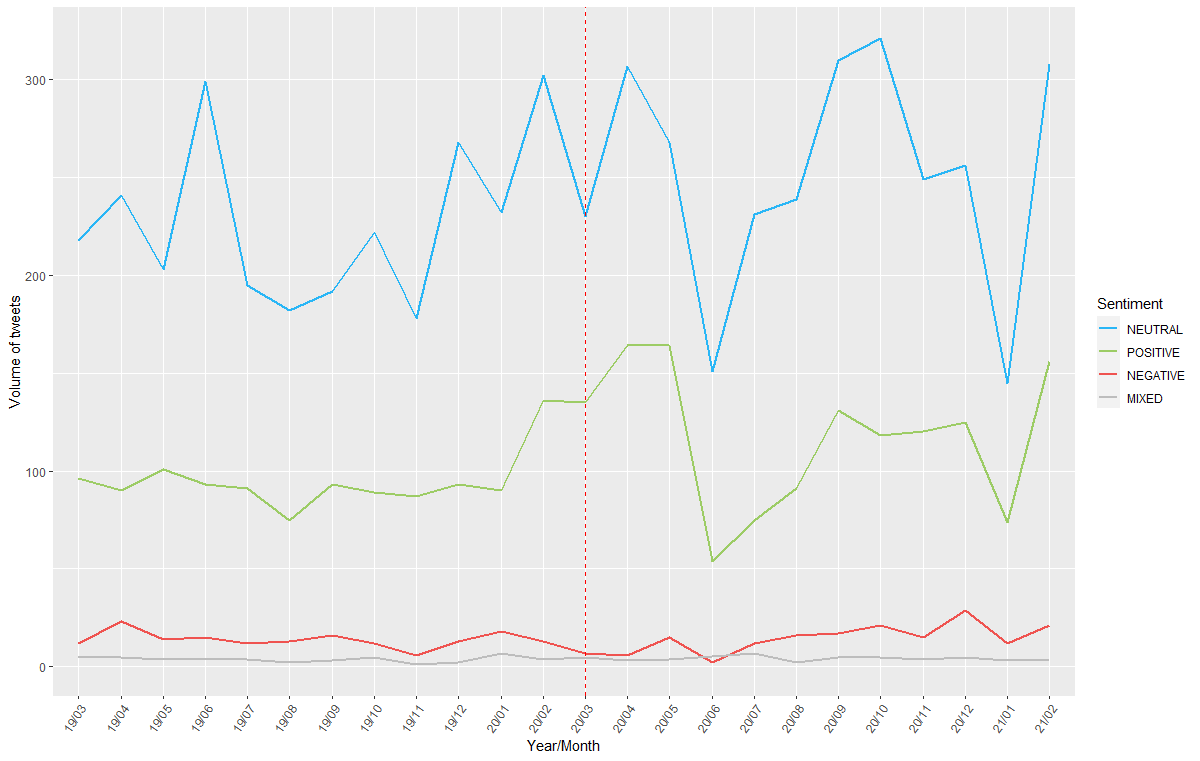

Supplement: S2 Fig — (PNG) [file pone.0313191.s002.png]
