# Supplementary material for: A social media competitive intelligence framework for brand topic identification and customer engagement prediction
Source: PLoS One. 2024 Nov 25;19(11):e0313191. doi: 10.1371/journal.pone.0313191 (PMC11588230; doi:10.1371/journal.pone.0313191)
Supplement: S1 Table — (DOCX) [file pone.0313191.s003.docx]

Appendix III. Labeled topics with descriptions and examples.

| **Situation** | | **Topic** | **Label** | **Description** | **Examples** |
| --- | --- | --- | --- | --- | --- |
| Normal | 1 | | Food and lifestyle | Connecting foods with peoples' lifestyle | Find someone you'll share pizza with for the rest of your life. |
|  |  |  |  |  | You spend 1/3 of your life asleep. The other two thirds is spent thinking about pizza. |
|  |  |  |  |  | Life is short. Eat dessert first. 🍫😜 https://x.xx/xxxxxxxxxxxxx |
|  | 2 | | Promotion | Ordinary promotions in social media | Was that a catch??? Either way, you can win incredible prizes during the next commercial break! #JacksTinyTacos |
|  |  |  |  |  | Blue...43...win free stuff! Tweet #JacksTinyTacos and #Giveaway before this commercial break ends for a chance at Prize Pack 10! https://t.co/xy2wIMFAtQ |
|  |  |  |  |  | Buy a regular freeze for $1, get a Doritos Locos Tacos for $1. Now that’s what we call #HappierHour. |
|  | 3 | | Food ordering | Calling customers to order foods and introducing channels of ordering | Ordering this in... 10 9 8 7 6 5 4 3 2 Done. https://t.co/6kjUYJS4FH |
|  |  |  |  |  | Should you order 🍍 pizza for #NationalPineappleDay? yes yes yes yes yes yes yes yes… |
|  |  |  |  |  | Cyber Monday is here DD Perks Members! So is your last chance to get 3X points on any food or beverage when you order using the Dunkin' App. Exclusions apply. https://t.co/nPaDb1SFAG |
|  | 4 | | Food time | Recalling customers' memory of approaching food time. | not sure who needed to hear this today, but it’s ok not to be happy all the time. all that matters is that you #FeelYourWay. https://t.co/vPmy1sT0cC https://t.co/XmF0GvMjCg |
|  |  |  |  |  | It's high time for a brownie 😉. https://t.co/b6rnR7PdT6 |
|  |  |  |  |  | Longer days mean more time for coffee. ☀️☕ |
|  | 5 | | Food delivery | Introducing the delivery service | Sometimes the bells mean surrender, other times it means free delivery has arrived. $10 minimum. Rules: https://t.co/HxyHjC8mxc |
|  |  |  |  |  | Spicy Nugg delivery knows no bounds @dmainy_13 https://t.co/yfILuE6FOh |
|  |  |  |  |  | Don’t leave work - just get your Chili’s delivered. You're welcome. #WednesdayWisdom |
| Pandemic | 1 | | Food time | Recalling customers' memory of approaching food time. | If I had a pizza for every time I talked about pizza I’d have a looooooooooooooooooooooooooooooooooooooooottttttttttttttttttttttttttttttt of pizza. |
|  |  |  |  |  | There’s👏no👏such👏thing👏as👏pizza👏time👏if👏pizza👏time👏is👏all👏the👏time👏 |
|  |  |  |  |  | Apps stand for appetizing right? Time to pizza-fy your phone for #NationalPizzaMonth. https://t.co/Wm80fxoN26 |
|  | 2 | | Coupons and offers | Giving codes of coupons and offering discount information | Let’s give a hearty round of applause for your favorite footlongs. $5 Footlongs when you buy 2 only in app/online. https://t.co/Vkbzoe6Vxj |
|  |  |  |  |  | Break bread for a lot less dough. Get $5 footlongs when you buy 2 in the app or online. https://t.co/m8XuKo9he5 |
|  |  |  |  |  | use code BOGO50 in the app or online for buy 1 get 1 50% off Footlongs. tag who you go splitsies with. |
|  | 3 | | Theme day for foods | Promoting related products on the theme day | 🎉 Wake up, it’s National Dunkin’ Day! 🎉 Give your brain a liquid hug and celebrate with a FREE medium hot or iced coffee with any purchase today, 9/29.🧡 Exclusions &amp; additional charges may apply. https://t.co/zMLm62jTRI |
|  |  |  |  |  | Your pup would appreciate a Big Mouth Bite to celebrate #NationalDogDay 🍔🐾🍔🐾 https://t.co/loygDHX0mR |
|  |  |  |  |  | We all love coffee in our own special way! Let us know how you enjoy a cup while celebrating #NationalCoffeeDay today. ☕️💚 What time of day do you need your coffee most? |
|  | 4 | | Social responsibility | Introducing the efforts in social responsibility | KFC is donating 621,240 pounds of food to food banks across the US through its Harvest Program to combat critical shortages created by COVID-19. With this donation, KFC will have provided 1.2 million pounds of food this year. Visit https://t.co/yJZvcSXDWV to learn more. https://t.co/4lGhzSUXo1 |
|  |  |  |  |  | We are nothing without Black lives.  There's no room for injustice. We commit to strengthening every facet of our culture and policies to foster an environment where equality for Black people is a priority. We'll use our platform to support this movement. #BlackLivesMatter |
|  |  |  |  |  | We remain committed to investing back in the communities we serve by supporting organizations dedicated to social justice, youth, and education in the Black community. |
|  | 5 | | Food and lifestyle | Connecting foods with peoples' lifestyle | You have food at home but it’s not pizza​ hint​ hint​ hint​ hint​ hint hint hint hint​ hint​ hint​ |
|  |  |  |  |  | Hmmm, wonder why your stomach just started rumbling. 🤔 https://t.co/x5LB0fwyLe |
|  |  |  |  |  | December is almost over, but there’s still PLENTY of time to get your pour on. After all of the festivities are over, take a breath and relax with the $5 Merry Berry ‘Rita. We think you’ve earned it. https://t.co/mC0kaqp7EF |
|  | 6 | | Brand speciality | Introducing specialty foods that represent a brand | Cheesecake is literally in our name. Does that mean we have really freakin' good cheesecake? Yes. But does it mean we have the best cheesecake you've ever had in your entire life? Also, yes. https://t.co/LTkHbLWEhw |
|  |  |  |  |  | The Chocolate Caramelicious Cheesecake made with Snickers® is good for when your sweet tooth has a sweet tooth. https://t.co/sJcplBAmxP |
|  |  |  |  |  | Got tunnel vision for Tiny Tacos? Our Jackmobile is pulling up soon to help our next fan Recover Harder #SuperJackdMonday https://t.co/kG2mCJ5vNe |
|  | 7 | | Warmth convoying | Convoying warmth and love | Is that love in the air? Or pepperoni? Try our Heart Shaped Pizza and a warm, chocolatey brownie. 🍫🍕 https://t.co/4N9DBxVKfG |
|  |  |  |  |  | I will be occupied this week with ................. my loved ones https://t.co/YWJrSCiDfY |
|  |  |  |  |  | We capital L.O.V.E you @TJOshie77. Please be our Valentine🏒💕 #LoveDunkin https://t.co/BrPvVNWC4u |
|  | 8 | | Calls to purchase | Persuading customers directly to purchase foods | We could all use a GroupNug. Come to the Wendy’s drive-thru today and get your free 4pc. nuggets! https://t.co/nCRGbFk4AO |
|  |  |  |  |  | Now more than ever, we could all use a free Doritos® Locos Tacos. Get yours this Tuesday 3/31 when you visit our drive-thru. |
|  |  |  |  |  | TODAY we’re giving away a new Flamin' Hot Doritos® Locos Tacos for FREE. Drive-thru and grab yours. |
|  | 9 | | News sharing | Sharing news around the world and the brands' own news | If @PostMalone was a #Pokémon which one would he be? https://t.co/kYAVTzJY9U |
|  |  |  |  |  | TEST YOUR BITE. 😬 Scan to unlock our Papadia Snapchat lens.🕹 https://t.co/GVOUa4twlX |
|  |  |  |  |  | Texas Roadhouse + Animal Crossing? 🤔🤔 |
|  | 10 | | Sense of taste | Estimating customers' perception of taste. | 🔥 A donut… but make it SPICY!🔥 Introducing the Spicy Ghost Pepper Donut ft. strawberry flavored icing with cayenne &amp; ghost pepper for a sweet heat treat at Dunkin’. 👻 🌶️ Grab one &amp; show your spicy side. 🔥🍩 https://t.co/dYgBp7eCMj |
|  |  |  |  |  | A cup of Costa Coffee is slow roasted for a delicious, smooth taste. Strength? We leave that decision to you. Find your preferred blend ☕️ https://t.co/FK3nOlSiOH https://t.co/oyKfmiYBFs |
|  |  |  |  |  | You won’t be able to resist falling for our Spicy Garlic Sauce. (See what we did there?)😏 https://t.co/FOhlcfkE9n |
|  | 11 | | Event promotion | Taking advantage of a specific event to promote | Share your best @WWE Superstar pose using #Sweepstakes and #PJxWWE, as we'll be hosting a #WrestleMania virtual watch party with #WWE. 5 fans will win FREE 🍕for a year along with a WWE Championship Replica Title Belt. Official rules: https://t.co/dxAQTToc3g https://t.co/mbDfh4Nbtw |
|  |  |  |  |  | #HappyHalloween! The tricks &amp; treats are on us this year &amp; we’ve got ONE MORE RIDDLE. Here’s your final clue. Find the riddle. Solve it. DM us the answer on Twitter for your chance to win $500 worth of Domino’s. 🍕 https://t.co/jaOmyWUI5N |
|  |  |  |  |  | Our favorite matchup of the year. Tune in to our #WrestleMania virtual watch party at 7pm EST #PJxWWE. https://t.co/7AVmTJYFo6 |
|  | 12 | | Contactless ordering and delivery | Highlighting the contactless ways of ordering and delivery during the COVID-19 pandemic | Contactless ordering for quick and easy pickup. Boom. https://t.co/z4H5Kvz6UF |
|  |  |  |  |  | Watching the contactless delivery arrive from the window like: *yay* 👁️👄👁️ |
|  |  |  |  |  | Mid-day refresh at your service. #DunkinMatcha Use the Dunkin’ App for a contactless way to order and pay ➡️ pick up via the drive-thru or carry-out. https://t.co/c3zrvcdV0A |
