# Supplementary material for: A social media competitive intelligence framework for brand topic identification and customer engagement prediction
Source: PLoS One. 2024 Nov 25;19(11):e0313191. doi: 10.1371/journal.pone.0313191 (PMC11588230; doi:10.1371/journal.pone.0313191)
Supplement: S2 Table — (DOCX) [file pone.0313191.s004.docx]

Appendix IV. Parameters in the model tuning

| **Classifier** | **Parameter** |
| --- | --- |
| SVM | NA |
| CART | NA |
| C5.0 | model = tree; trial = [1, 10, 20, 30, 40, 50, 60, 70, 80, 90, 100]; winnow = [TRUE, FALSE] |
| Random forest | mtry = [3,6,9] |
| Bagged CART | NA |
| Gradient boosting | shrinkage = 0.1;  interaction depth = [1; 3; 5]; number of minobsinnode = [2; 5; 10]; number of trees = [100; 200; 300] |
