# Supplementary material for: A social media competitive intelligence framework for brand topic identification and customer engagement prediction
Source: PLoS One. 2024 Nov 25;19(11):e0313191. doi: 10.1371/journal.pone.0313191 (PMC11588230; doi:10.1371/journal.pone.0313191)
Supplement: S3 Table — (DOCX) [file pone.0313191.s005.docx]

Appendix V. Descriptive statistics of the categorical variables used in the predictive modeling.

| **Variable** | **Class** | **Frequency** | **Percentage** |
| --- | --- | --- | --- |
| Food type | Burger/Sandwich | 1072 | 28.37% |
|  | Casual dining | 683 | 18.08% |
|  | Coffee/Dessert | 526 | 13.92% |
|  | Fried chicken | 277 | 7.33% |
|  | Pizza/Italian food | 848 | 22.45% |
|  | Taco/Mexican food | 372 | 9.85% |
| Published time of tweet | Morning | 922 | 24.40% |
|  | Afternoon | 2308 | 61.09% |
|  | Night | 548 | 14.51% |
| Published day of tweet | Monday | 592 | 15.67% |
|  | Tuesday | 640 | 16.94% |
|  | Wednesday | 603 | 15.96% |
|  | Thursday | 603 | 15.96% |
|  | Friday | 583 | 15.43% |
|  | Saturday | 370 | 9.79% |
|  | Sunday | 387 | 10.24% |
| Media type | No media | 1805 | 47.78% |
|  | Image | 1385 | 36.66% |
|  | Gif | 49 | 1.30% |
|  | Video | 539 | 14.27% |
| Sentiment | NEUTRAL | 2505 | 66.30% |
|  | POSITIVE | 1107 | 29.30% |
|  | NEGATIVE | 123 | 3.26% |
|  | MIXED | 43 | 1.14% |
| Topic | Food and lifestyle | 574 | 15.19% |
|  | Promotion | 504 | 13.34% |
|  | Food ordering | 576 | 15.25% |
|  | Food time | 747 | 19.77% |
|  | Food delivery | 429 | 11.36% |
|  | Coupons and offers | 72 | 1.91% |
|  | Theme day for foods | 117 | 3.10% |
|  | Social responsibility | 143 | 3.79% |
|  | Brand speciality | 60 | 1.59% |
|  | Warmth convoying | 74 | 1.96% |
|  | Calls to purchase | 77 | 2.04% |
|  | News sharing | 86 | 2.28% |
|  | Sense of taste | 92 | 2.44% |
|  | Event promotion | 112 | 2.96% |
|  | Contactless ordering and delivery | 115 | 3.04% |
| Customer engagement | Likes < 194 | 1956 | 51.77% |
|  | Likes >= 194 | 1822 | 48.23% |
|  | Reply < 24 | 1958 | 51.83% |
|  | Reply >= 24 | 1820 | 48.17% |
|  | Retweets < 25 | 1905 | 50.42% |
|  | Retweets >= 25 | 1873 | 49.58% |
|  | Sum | 3778 | 100.00% |
