# Supplementary material for: A social media competitive intelligence framework for brand topic identification and customer engagement prediction
Source: PLoS One. 2024 Nov 25;19(11):e0313191. doi: 10.1371/journal.pone.0313191 (PMC11588230; doi:10.1371/journal.pone.0313191)
Supplement: S4 Table — (DOCX) [file pone.0313191.s006.docx]

Appendix VI. Descriptive statistics of the continuous variables used in the predictive modeling.

| **Variable** | **Min** | **Q1** | **Median** | **Mean** | **Q3** | **Max** |
| --- | --- | --- | --- | --- | --- | --- |
| Number of mentions | 0 | 0 | 0 | 0.22 | 0 | 10 |
| Number of hash Tag | 0 | 0 | 0 | 0.4 | 1 | 7 |
| Number of links | 0 | 0 | 1 | 0.82 | 1 | 4 |
| Length of tweets | 1 | 13 | 20 | 23.94 | 33 | 119 |
| Readbility of tweets | -7.84 | 6.08 | 9.83 | 10.32 | 13.69 | 63.28 |
| COVID-19 infected numbers | 0 | 0 | 0 | 22383.47 | 26428 | 257750 |
| COVID-19 death tolls | 0 | 0 | 0 | 449.23 | 717 | 5061 |
